# Impact of lower co-payments on risk-reducing salpingo-oophorectomy and *BRCA* testing in Japan

**DOI:** 10.1186/s13690-023-01048-9

**Published:** 2023-02-27

**Authors:** Katsuyuki Konnai, Hiroyuki Fujiwara, Masakazu Kitagawa, Reina Wakabayashi, Asuna Yumori, Tsuguto Notomi, Ryo Onose, Hisamori Kato, Hiroto Narimatsu

**Affiliations:** 1 Department of Gynecology, Kanagawa Cancer Center, 2-3-2 Nakao, Asahi-ku, Yokohama, Kanagawa 241-8515 Japan; 2 Department of Obstetrics and Gynecology, Jichi Medical University, Tochigi, Japan; 3 Yamato Municipal Hospital, Yamato, Kanagawa Japan; 4 Cancer Prevention and Control Division, Kanagawa Cancer Center Research Institute, Yokohama, Kanagawa Japan

**Keywords:** *BRCA* testing, Health insurance, Hereditary breast and ovarian cancer, Preventive medical service, Price elasticity, Risk-reducing salpingo-oophorectomy

## Abstract

**Background:**

In April 2020, insurance coverage for risk-reducing salpingo-oophorectomy (RRSO) for breast cancer patients with hereditary breast and ovarian cancer (HBOC) syndrome and *BRCA* testing were started in Japan. We investigated the impact of insurance coverage on the number of RRSO and *BRCA* tests performed.

**Methods:**

The subjects were 370 breast cancer patients and 23 of their relatives who received genetic counseling at our institution between April 2014 and December 2021. Finally, 349 patients and 15 relatives were analyzed. We retrospectively compared the number of *BRCA* tests, RRSO, insurance status, and co-payment of medical expenses before and after insurance coverage based on medical records.

**Results:**

In the 6-year pre-coverage period, 226 patients (mean: 37/year) received genetic counseling and 106 (17/year) received *BRCA* testing. In the 21-month post-coverage period, 161 patients (92/year) received genetic counseling and 127 (72/year) received *BRCA* testing. The rate of testing/counseling significantly increased in the post-coverage period (46.9% vs. 78.8%; *p* < .001). The number of patients who were diagnosed with HBOC were 24 (4/year) and 18 (10/year) and RRSO was performed for 7 (1/year) and 11 (6/year) patients in the pre- and post-coverage periods, respectively. The rate of RRSO/HBOC was significantly increased in the post-coverage period (29.1% vs. 61.1%; *p* = 0.039). RRSO patients' co-payment rates decreased from 64% to 25% pre- and post-coverage.

**Conclusions:**

Our findings suggest that decreased co-payments were the primary reason for these increases. Insurance coverage is an important factor when promoting preventive medical services such as RRSO.

## Background

Hereditary breast and ovarian cancer (HBOC) syndrome is associated with an increased cumulative risk of breast and ovarian cancer [1]. Risk-reducing salpingo-oophorectomy (RRSO) reduces the relative risk of ovarian cancer by about 80% [2] and breast cancer by about 40% in woman with *BRCA1*/*2* mutations who had no history of breast cancer [3]. Moreover, RRSO has been reported to significantly reduce the incidence of high-grade serous carcinoma [4]. RRSO is typically recommended for patients aged between 35 and 40 years and upon completion of childbearing for breast cancer patients with *BRCA1* pathological variants, and between 40 and 45 years for *BRCA2* pathological variants [5]. In a nationwide multicenter study of *BRCA* mutation prevalence in ovarian, fallopian tube, and peritoneal cancers in Japan, the most common histological type was high-grade serous carcinoma, with a prevalence of 28.5% [6].

Japan's medical insurance system is a universal health insurance system that covers all citizens with public medical insurance. Patients are free to choose the medical institutions and receive equal medical care. Patients under the age of 70 pay a 30% co-payment in principle. The co-payment is set lower for infants and those over 70 years old. In addition, high-cost medical expense benefit system reimbursement is available. In Japan, RRSO was introduced around 2008 and was not covered by insurance [7]. Moreover, *BRCA* germline testing, which is a hereditary cancer test for HBOC, was also not covered by insurance. However, since July 2018, *BRCA* testing has been partly covered by insurance for recurrent breast cancer patients as a companion diagnosis for olaparib. From April 2020, it was expanded for breast cancer patients including those under 45 years of age, triple-negative breast cancer patients under 60 years of age, two or more primary tumors, at least one person with breast or ovarian cancer within third-degree relatives, and male breast cancer. RRSO became covered for breast cancer patients with *BRCA* pathological variants [8].

The number of *BRCA* tests and RRSO have been increasing after these procedures were partly covered by insurance; however, it is unclear how the trends of RRSO and *BRCA* tests have changed as a result.

To evaluate the impact of insurance coverage on the decision of breast cancer patients with HBOC syndrome to undergo RRSO, we investigated the change in the number of patients and co-payment before and after insurance coverage.

## Methods

**Fig. 1:**
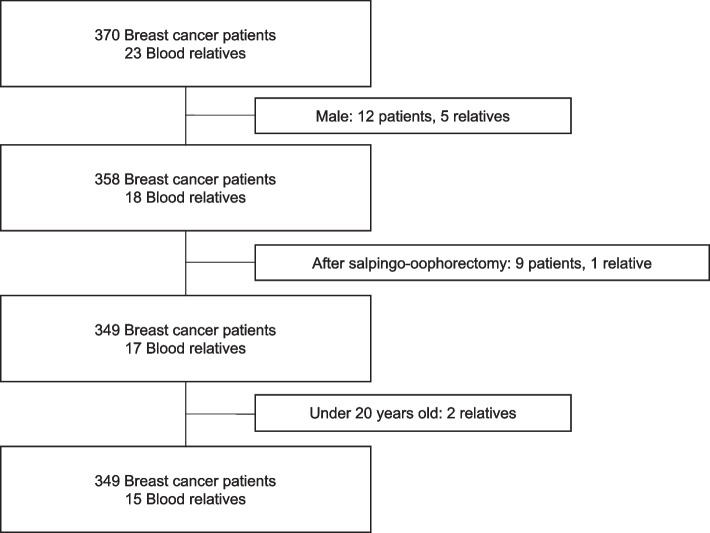
Flowchart of study selection. The subjects were 370 breast cancer patients and 23 of their relatives who received genetic counseling at Kanagawa Cancer Center between April 2014 and December 2021. Finally, 349 patients and 15 relatives were analyzed

The subjects were 370 breast cancer patients and 23 of their relatives who received genetic counseling at our institution between April 2014 and December 2021. Women over 20 years old whose ovaries and fallopian tubes had not been removed were eligible. Finally, 349 patients and 15 relatives were analyzed (Fig. 1).

We divided the patients into two groups: a pre-coverage group for cases performed during a 6-year period from April 2014 to March 2020, and a post-coverage group for cases performed during the 21-month period from April 2020 to December 2021. Between the two groups, the number of *BRCA* germline tests and RRSO performed, insurance status, and co-payment of medical expenses were retrospectively compared using medical records.

In the pre-coverage group, if a breast cancer patient with a *BRCA1* or *BRCA2* pathological variant wished to undergo RRSO, the treatment plan was discussed at a conference consisting of a gynecologic oncologist, breast surgeon, clinical geneticist, and certified genetic counselor, and was approved by the Ethics Committee. If uterine fibroids or ovarian tumors were present, hysterectomy and salpingo-oophorectomy were performed under health insurance.

In the post-coverage group, RRSO costs were covered by insurance. If a hysterectomy was performed during RRSO, the hysterectomy was 100% self-pay unless a lesion was identified in the uterus. In such a case, as in the case of a fracture during hospitalization for childbirth, mixed medical treatment is not applicable.

Pathological examination of the removed fallopian tubes was performed by the Sectioning and Extensively Examining the Fimbria protocol. The fallopian tubes were sectioned at 2-3 mm intervals, and the distal 2 cm of the tube was amputated and sectioned sagittally into four sections [9].

The statistical software used was IBM SPSS Version 20 for Windows (IBM, USA). T-test and Fisher's exact test were used to compare before and after insurance coverage. For the comparison of the number of *BRCA* tests and RRSO, one-sided *p*<0.05 was considered significant, and the other parameters were two-sided.

## Results

### Clinical background

The breast cancer patients' clinical background is presented in Table 1. There were no significant differences in age at diagnosis, age at counseling, hormone sensitivity type, number of bilateral cancer, family history, and parity between the two groups.

**Table 1:** Clinical background of breast cancer patients

	Pre-coverage period (6 years) *N*=207	Post-coverage period (1 year 9 months) *N*=142	p
Age at diagnosis, mean (SD)	47 (12.0)	48 (10.4)	0.66^*^
Age at counseling, mean (SD)	51 (12.1)	51 (12.1)	0.78^*^
Hormone sensitivity type			
Luminal (%)	151 (73.0)	102 (71.8)	0.90^‡^
Triple negative (%)	38 (18.3)	25 (17.6)	0.88^‡^
HER2 (%)	12 (5.8)	13 (9.2)	0.29^‡^
Unknown (%)	6 (2.9)	2 (1.4)	
Bilateral	45	33	0.79^‡^
metachronous	28	20	
synchronous	17	13	
Family history			0.79^‡^
Yes	162	109	
No	45	33	
Parity			0.80^‡^
Yes	154	104	
No	53	38	

^*^t-test

^‡^Fisher's exact test

### Number of *BRCA* tests

**Fig. 2:**
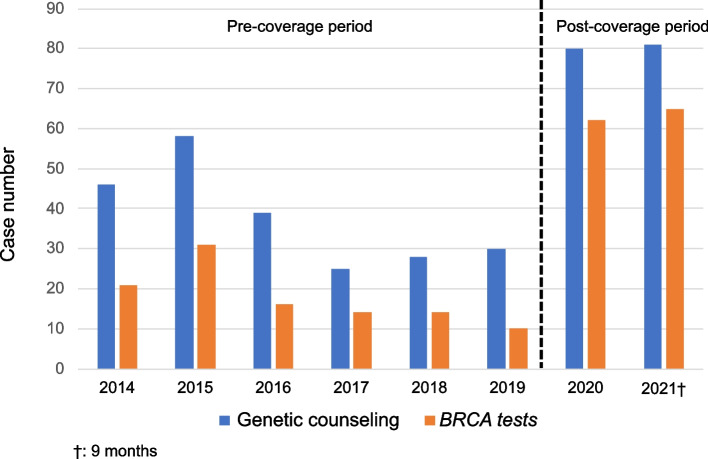
Annual change of genetic counseling and *BRCA* tests. In the pre-coverage period, 226 patients (37/year) received genetic counseling and 106 (17/year) received *BRCA* testing. In the post-coverage period, 161 patients (92/year) received genetic counseling and 127 (72/year) received *BRCA* testing

A total of 328 breast cancer patients and 14 blood relatives received one counseling, and 21 breast cancer patients and one blood relative received multiple counseling. In the 6-year pre-coverage period (2014-2019), 226 patients (mean: 37/year) received genetic counseling and 106 (17/year) received *BRCA* testing. During this period *BRCA* testing was covered by insurance for eight patients, whereas all the rest were self-pay. In the 21-month post-coverage period (2020-2021), 161 patients (92/year) received genetic counseling and 127 (72/year) received *BRCA* testing. Of the 127 that received *BRCA* testing, 118 were covered by insurance and nine at self-pay (asymptomatic blood relatives and multi-gene panel test). The rate of testing/counseling significantly increased in the post-coverage period (46.9% vs. 78.8%; *p* < 0.001) (Table 2, Fig. 2).

**Table 2:** Number of patients that received genetic counseling and *BRCA* tests

	Pre-coverage period (6 years)	Post-coverage period (1 year 9 months)	p^*^
Number of genetic counseling (mean)	226^a^ (37/year)	161^a^ (92/year)	
Number of *BRCA* tests (mean)	106 (17/year)	127 (72/year)	
Ratio of testing/counseling	46.9%	78.8%	<0.001

^*^Fisher's exact test

^a^Twenty-one breast cancer patients and one blood relative received multiple counseling

### Number of RRSO

**Fig. 3:**
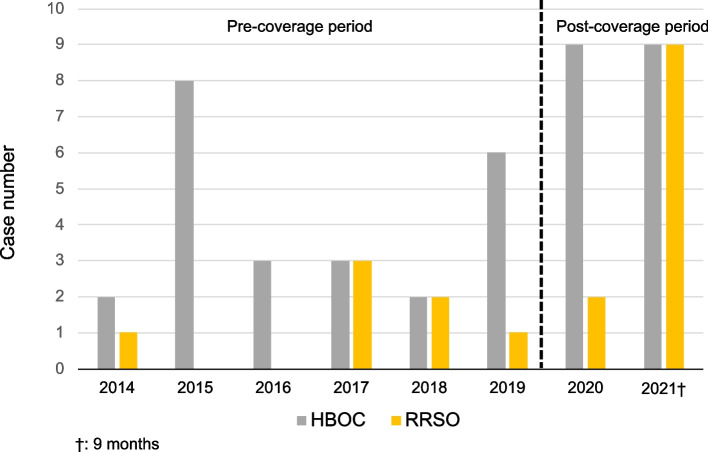
Annual change of HBOC and RRSO cases. The number of patients who were diagnosed with HBOC were 24 (4/year) and 18 (10/year) and RRSO was performed for 7 (1/year) and 11 (6/year) patients in the pre- and post-coverage periods, respectively. HBOC: hereditary breast and ovarian cancer, RRSO: risk-reducing salpingo-oophorectomy

Twenty-four patients (4/year) were diagnosed with HBOC in the pre-coverage period, and 18 (10/year) in the post-coverage period. RRSO was performed on 18 patients for the entire period. There were seven patients (1/year) in the pre-coverage period and 11 patients (6/year) in the post-coverage period. The rate of RRSO/HBOC significantly increased in the post-coverage period (29.1% vs. 61.1%; *p* = 0.039) (Table 3, Fig. 3).

**Table 3:** Number of patients diagnosed with HBOC and received RRSO

	Pre-coverage period (6 years)	Post-coverage period (1 year 9 months)	p^*^
Number of HBOC (mean)	24 (4/year)	18 (10/year)	
Number of RRSO (mean)	7 (1/year)	11 (6/year)	
Ratio of RRSO/HBOC	29.1%	61.1%	<0.05

*HBOC* hereditary breast and ovarian cancer, *RRSO* risk-reducing salpingo-oophorectomy

^*^Fisher's exact test

The case list is shown in Table 4. Two patients in the pre-coverage group and four patients in the post-coverage group had *BRCA1* pathological variants, and five patients and seven patients had *BRCA2* in the pre-coverage and the post-coverage groups, respectively. Hysterectomy was performed in five of six *BRCA1* pathological variants (Patient 1 of the *BRCA1* pathological variant group had already had a hysterectomy due to a history of uterine fibroids) and 5 of 12 *BRCA2* pathological variants. Four out of seven patients in the pre-coverage group were 100% self-pay. The other three patients had abnormal findings in the uterus and adnexa; therefore, they were covered by insurance. In the post-coverage group, 8 of 11 patients were covered by insurance, and in the other three patients, RRSO was covered by insurance and hysterectomy was performed at 100% self-pay due to normal findings of the uterus. The average medical expenses of laparoscopic bilateral salpingo-oophorectomy (LBSO) was 599,653 JPY (4,222 USD [1 USD = 142 JPY]) and the co-payment was 68,557 JPY (482 USD) in the post-coverage group. Moreover, total laparoscopic hysterectomy (TLH) + LBSO was 1,085,542 JPY (7,644 USD) and 534,843 JPY (3,766 USD), respectively. The average co-payment ratio (Co-payment/Medical expenses) were 64% and 25% in the pre- and post-coverage groups, respectively. Serous tubal intraepithelial carcinoma was detected in one patient with *BRCA2* pathological variant.

**Table 4:** RRSO case list

	Case number	Year	Age	*BRCA1/2*	Surgical procedure	Insurance/Self-pay	Medical expenses (JPY)	Co-payment (JPY)	Co-payment ratio (%)	STIC
Pre-coverage group	1	2014	65	*1*	ABSO+CRRM	Self-pay	1,025,930	1,025,930	100	No
	2	2017	47	*2*	TAH+ABSO	Insurance	845,390	0^a^	0	No
	3	2017	50	*2*	TLH+LBSO	Self-pay	948,190	948,190	100	Yes
	4	2017	44	*2*	TLH+LBSO	Insurance	880,134	264,040	30	No
	5	2018	60	*2*	LBSO	Self-pay	660,000	660,000	100	No
	6	2018	49	*2*	TAH+ABSO	Insurance	873,940	178,839	20	No
	7	2019	45	*1*	TLH+LBSO	Self-pay	1,189,062	1,189,062	100	No
Post-coverage group	8	2020	64	*1*	TLH+LBSO	Insurance+Self-pay	1,136,060	668,280	59	No
	9	2020	31	*2*	LBSO	Insurance	531,420	59,900	11	No
	10	2021	57	*2*	LBSO	Insurance	602,060	59,900	10	No
	11	2021	57	*2*	LBSO	Insurance	614,120	59,900	10	No
	12	2021	53	*2*	TLH+LBSO+LA	Insurance	1,172,870	179,989	15	No
	13	2021	54	*2*	LBSO	Insurance	613,550	85,866	14	No
	14	2021	42	*1*	TLH+LBSO	Insurance+Self-pay	1,119,960	663,382	59	No
	15	2021	72	*2*	LBSO	Insurance	622,110	59,900	10	No
	16	2021	59	*2*	LBSO	Insurance	614,660	85,877	14	No
	17	2021	52	*1*	TLH+LBSO	Insurance	951,180	257,372	27	No
	18	2021	48	*1*	TLH+LBSO	Insurance+Self-pay	1,130,610	550,341	49	No

*STIC* serous tubal intraepithelial carcinoma, *ABSO* abdominal bilateral salpingo-oophorectomy, *CRRM* contralateral risk-reducing mastectomy, *TAH* total abdominal hysterectomy, *TLH* total laparoscopic hysterectomy, *LBSO* laparoscopic bilateral salpingo-oophorectomy, *LA* laparoscopic appendectomy

^a^The co-payment was zero for low-income patients

## Discussion

In the present study, we found that both the number of RRSO and *BRCA* tests increased significantly after insurance part coverage was extended. The number of RRSO in the 21-month period after April 2020, when health insurance partly coverage was started, exceeded the 6-year period before coverage. The number of *BRCA* tests for diagnosing HBOC also increased. Figure 3 shows a notable difference in the HBOC/RRSO ratio between 2020 and 2021. This indicates that the decision for RRSO was not made as soon as the diagnosis of HBOC was confirmed. Breast cancer patients sometimes undergo *BRCA* testing to determine the surgical procedure. In the case of HBOC, total mastectomy is recommended. Therefore, patients tend to undergo *BRCA* testing prior to breast cancer surgery and then undergo RRSO once they are undergoing breast cancer treatment.

In Japan, for procedures covered by insurance, the co-payment is 30% of the total cost in principle. The cost of the *BRCA* testing is approximately 200,000 JPY (1,408 USD), so the co-payment is 60,000 JPY (422 USD), covered by insurance. In the case of laparoscopic RRSO (=LBSO), medical expenses are about 600,000 JPY (4,225 USD); thus, the co-payment is about 180,000 JPY (1,267 USD). However, actual co-payments were lower, from 59,900 (421 USD) to 85,877 JPY (604 USD), and the co-payment ratio was only 10-14% as these patients were covered by the High-cost Medical Expense Benefit system, which allows patients to limit the amount of co-payment to a fixed monthly amount by applying in advance. The maximum amount of the co-payment is determined according to the patient's age and income. This system only applies to medical expenses that are covered by insurance.

Our findings suggest that the increase in the number of RRSO and *BRCA* tests was due to the decrease in co-payment. This phenomenon can be explained by price elasticity (PE). PE is a measure of how much the consumption of a product or service changes with changes in price, which is expressed as the percent change in consumption for a 1% change in price.

$$\textrm{PE}=\frac{\varDelta \textrm{q}/\textrm{q}}{\varDelta \textrm{p}/\textrm{p}}=\frac{\textrm{Percent}\ \textrm{change}\ \textrm{in}\ \textrm{quantity}\ \textrm{demanded}\ \textrm{of}\ \textrm{good}\ \textrm{A}}{\textrm{Percent}\ \textrm{change}\ \textrm{in}\ \textrm{price}\ \textrm{of}\ \textrm{good}\ \textrm{A}}$$

This tool is useful because it is a unitless measure and provides information about the nature of the product (necessity or luxury) and the relationship between the products (substitutes or complements): if the absolute value of PE is less than 1, it is considered price inelastic; if it is greater than 1, it is considered price elastic [10].

PE tends to center on -0.17 in health care [11]. This indicates that a 1% increase in price results in a 0.17% decrease in demand. In general, services in health care are considered to be price inelastic; thus, there is little decrease in demand due to price increases. However, preventive care and pharmacy benefits are among the medical services with larger PE. When the price of care increases, consumers are able to substitute away from preventive care toward other goods and services that promote health such as nutritional supplements and healthy foods. In addition, preventive medical services may be put off when the price of such care increases [11]. RRSO is preventive care; thus, it may have been treated as a luxury. For *BRCA1/2* pathogenic variant carriers, the risk of ovarian cancer, which is known for its very poor prognosis, is high. Furthermore, reliable screening methods to detect early-stage ovarian cancer are unavailable [12]. Our findings suggest that RRSO should not be considered a luxury item because it can reliably reduce the risk of ovarian cancer.

Price has been reported to be an important factor for health care promoters. In a study conducted in Sudan, free use of health centers for malaria treatment at different levels of coverage (25%, 50%, and 75%) increased their use for the treatment of children to 63.6%, 32.3%, and 280.4%, respectively (PE was -2.5, -0.6, and -3.7, respectively) [13]. In 2004, German health authorities introduced a 50% co-payment for patients, in an effort to save the cost of *in-vitro* fertilization (IVF) and intracytoplasmic sperm injection (ICSI). Prior to that, 100% reimbursement was available for up to four IVF and ICSI cycles. After the co-payment increase, the number of IVF and ICSI cycles decreased by 53% [14]. In Korea, *BRCA* testing and RRSO have been covered under the Korean universal health insurance system since 2012, and the number of both procedures has been increasing every year. The expansion of insurance coverage is thought to be one of the factors for this increase [15].

In this study, the number of RRSO increased 5.4 times after the procedure was covered by insurance. RRSO decreased from 100% self-payment to 30%, and the PE was -7.7. Similarly, the PE of the *BRCA* testing was -5.8. The PE of both was quite high as PE is considered in absolute value and price declines are considered the main factor in high PE. In addition, the establishment of the Japanese Organization of Hereditary Breast and Ovarian Cancer (2016), the publication of the Guidebook for Diagnosis and Treatment of HBOC Syndrome (2017), and Guidelines for Diagnosis and Treatment of HBOC syndrome (2021) improved the understanding of breast surgeons, gynecologists, and other medical professionals. Moreover, breast cancer patients have access to more information about HBOC syndrome. Price is reported to be more likely to influence an individual's decision to receive treatment than the frequency of visits after receiving treatment [16]; thus, it may influence the decision to receive RRSO.

In Japan, all medical care for asymptomatic blood relatives of HBOC syndrome is not covered by insurance. The frequency of *BRCA1/2* pathological variant in the general population in Japan is reported to be 0.21% [17], which is relatively high. In the future, we expect insurance coverage for medical care for asymptomatic blood relatives. Medical treatment of HBOC syndromes, such as hypertension and hyperlipidemia, is a preventive intervention; thus, we propose that it should be covered by insurance. Because, at present, ovarian cancer cannot be reliably detected in its early stages by screening. Several reports indicated that RRSO is more cost-effective than surveillance [18, 19]. In the U.S., health care coverage for the working-age population is provided primarily through private health insurance, with major insurance companies covering *BRCA* testing and RRSO. Because this is the least expensive method for managing the insured’s risk [20]. In addition, RRSO is covered by insurance in Korea regardless of whether the patient has breast cancer or not. *BRCA* testing is also covered by insurance and can be performed in-house; thus, the co-payment is less than 100 USD [21].

In this study, only one patient who underwent RRSO was within the recommended age range according to the current guidelines and most patients were older. The same trend was observed in a previous report that analyzed national-scale data for Japan [22]. The reason may be that most people underwent genetic testing after they developed breast cancer. In the future, the average age of RRSO may decrease if insurance coverage is extended to asymptomatic HBOCs and they undergo genetic testing. Patient number nine was younger but had a 58-mm bifid tumor on her left ovary and she had two children; thus, she strongly desired to remove the tumor.

Hysterectomy at the time of RRSO was performed in all cases with the *BRCA1* pathological variant, except for the post-hysterectomy case. We speculate that the reason for this is that information was provided to patients before performing the RRSO. A previous analysis of 1083 women who underwent RRSO without hysterectomy revealed that although the overall risk for uterine cancer after RRSO was not increased, the risk for serous/serous-like endometrial carcinoma was increased in *BRCA1* pathogenic women [23]. According to a report by the Japan Society of Obstetrics and Gynecology, the overall 5-year survival rate is good for G1 and G2 endometrioid carcinomas (96.7% and 88.3%, respectively), whereas it is poor for serous endometrial carcinoma (60.9%) [24]. In the case with *BRCA1* pathogenic variant, the patient desired a hysterectomy. The composition of hormone replacement therapy was not an argument for hysterectomy in this study. RRSO was covered by insurance, but if there was no abnormality in the uterus, hysterectomy was self-pay, and its cost was approximately 460,000 JPY (3,239 USD). It is important to explain the advantages and disadvantages of hysterectomy before surgery.

This study had three limitations. First, the number of cases was limited because the study was conducted at a single institution. Our institution is the only cancer center in Kanagawa Prefecture, which is the second most populous region in Japan [25]. As other factors were stable in the study period, the results of our institution may reflect the influence of the insurance coverage. Second, the period of time after insurance coverage was relatively short compared with the pre-coverage period. However, the number and acceptance rate of RRSO and *BRCA* testing increased after insurance coverage; thus, we evaluated the influence of insurance coverage at this point. Third, insurance coverage may have had a potential influence on the counseling of the clinical geneticist; however, this was not evaluated in the present study.

## Conclusion

This study found that the number of RRSO and *BRCA* tests increased significantly after insurance coverage. *BRCA* testing and RRSO are price elastic, and decreased co-payments were suggested as the primary reason for the increase in the number of both procedures. Thus, insurance coverage is an important factor when promoting preventive medical services such as RRSO.

## Data Availability

All data generated or analysed during this study are included in this published article.

## Abbreviations

RRSO: Risk-reducing salpingo-oophorectomy
HBOC: Hereditary breast and ovarian cancer
LBSO: Laparoscopic bilateral salpingo-oophorectomy
TLH: Total laparoscopic hysterectomy
PE: Price elasticity
IVF: *in-vitro* fertilization
ICSI: Intracytoplasmic sperm injection
